# Molecular Defensive Mechanism of *Echinacea purpurea* (L.) Moench against PAH Contaminations

**DOI:** 10.3390/ijms241311020

**Published:** 2023-07-03

**Authors:** Caixia Sun, Xiangbo Shen, Yulan Zhang, Tianshu Song, Lingjing Xu, Junyao Xiao

**Affiliations:** 1 Key Laboratory of Bioresource Research and Development of Liaoning Province, College of Life and Health Sciences, Northeastern University, Shenyang 110169, China; 2 Liaoning Province Outstanding Innovation Team, Institute of Applied Ecology, Chinese Academy of Sciences, Shenyang 110016, China

**Keywords:** polycyclic aromatic hydrocarbons, phytoremediation, transcriptomics, metabolomics

## Abstract

The understanding of the molecular defensive mechanism of *Echinacea purpurea* (L.) Moench against polycyclic aromatic hydrocarbon (PAH) contamination plays a key role in the further improvement of phytoremediation efficiency. Here, the responses of *E. purpurea* to a defined mixture of phenanthrene (PHE) and pyrene (PYR) at different concentrations or a natural mixture from an oilfield site with a history of several decades were studied based on transcriptomics sequencing and widely targeted metabolomics approaches. The results showed that upon 60-day PAH exposure, the growth of *E. purpurea* in terms of biomass (*p* < 0.01) and leaf area per plant (*p* < 0.05) was negatively correlated with total PAH concentration and significantly reduced at high PAH level. The majority of genes were switched on and metabolites were accumulated after exposure to PHE + PYR, but a larger set of genes (3964) or metabolites (208) showed a response to a natural PAH mixture in *E. purpurea*. The expression of genes involved in the pathways, such as chlorophyll cycle and degradation, circadian rhythm, jasmonic acid signaling, and starch and sucrose metabolism, was remarkably regulated, enhancing the ability of *E. purpurea* to adapt to PAH exposure. Tightly associated with transcriptional regulation, metabolites mainly including sugars and secondary metabolites, especially those produced via the phenylpropanoid pathway, such as coumarins, flavonoids, and their derivatives, were increased to fortify the adaptation of *E. purpurea* to PAH contamination. These results suggest that *E. purpurea* has a positive defense mechanism against PAHs, which opens new avenues for the research of phytoremediation mechanism and improvement of phytoremediation efficiency via a mechanism-based strategy.

## 1. Introduction

Polycyclic aromatic hydrocarbons (PAHs) are a family of environmentally persistent, widely distributed organic pollutants produced by the incomplete combustion of carbon-based fuels or originating from the release of petroleum into the environment [1,2]. These substances are readily adsorbed by soil particles and highly resistant to degradation by natural mechanisms, which cause their accumulation in the soil ecosystem and consequent entry into the human food chain [3]. Given their acute toxicity, carcinogenicity, mutagenicity, and teratogenicity, the remediation of PAH contamination is an ongoing endeavor. Among diverse options for remediation of PAHs, phytoremediation, a method that utilizes suitable plants to remove PAHs from contaminated environments, is a cost-effective and environmentally friendly strategy that has considerable advantages, such as low operating costs, non-interference with polluted sites and ecosystems, and the possibility to clean up different pollutions concomitantly [4,5,6].

The success of PAH phytoremediation is largely determined by the selection of suitable plant candidates because phytoremediation efficiency varies remarkably among plant species [7]. Numerous plant species, such as aquatic weeds, grasses, sedges, legumes, and trees, have been verified as strong candidates for crude oil and/or PAH remediation [8,9,10]. *E. purpurea*, an Asteraceae family ornamental plant, has attracted particular interest in the phytoremediation of PAH soil contamination in the past decade [11,12,13]. *E. purpurea* can survive under various soil conditions and stresses, such as saline, drought, and cold, owing to its highly developed fibrous root system, strong growth, and large biomass [14]. PAH phytoremediation with *E. purpurea* has further advantages in revegetation cover and beautification of green spaces [13,15]. PAH detoxification in plants is a multi-enzyme multi-step process, and a three-phase transformation model for xenobiotics has been proposed [16,17]. However, information about the molecular regulation and metabolic responses of *E. purpurea* to PAH stress is still lacking, which limits the further improvement of phytoremediation efficiency via a mechanism-based strategy. Through providing the whole-genome transcriptional profiling of plants, transcriptome sequencing has increased our understanding of the pathways/processes perturbed by exposure to phenanthrene (PHE) or benzo[a]pyrene (BaP) pollution in *Arabidopsis thaliana* (L.) and *Ulva lactuca* (L.) [18,19,20]. Untargeted metabolomics has been used to explore biochemical modulations, such as sugar metabolism and amino acid biosynthesis, in response to BaP and pyrene (PYR) or PHE exposure [7,19]. As a sensitive and accurate system based on the measurement of targeted metabolites, widely targeted metabolomics was used to explore the adaptive response of *Salix viminalis* (L.) to PHE [21]. Therefore, the integration of the transcriptomic data with metabolomic data will greatly help in elucidating the phytoremediation mechanisms and identifying novel target genes and metabolites for the enhancement of plant capability to metabolize PAHs.

As model PAHs, PHE and PYR have gained attention and are extensively studied worldwide owing to their typical structure, property, and predominance in agricultural soil [2,22]. In phytoremediation research, the intricacies of mixture contamination need to be considered to develop phytoremediation strategies for real contaminated sites where PAHs are present as mixtures rather than single chemicals [23,24,25]. In addition, the physiological responses of plants are dependent on PAH concentration (content) or intensity (toxicity) [6,26]. Therefore, two approaches, namely, a component-based approach (defined mixtures of two PAHs, PHE and PYR, at three concentrations) or a natural mixture approach (a natural mixture of PAHs from oilfield-contaminated soils), were designed to avoid the underestimation of the complexity and heterogeneity of the real polluted environment.

Here, the main objectives were to (1) identify candidate genes, metabolite targets, and pathways/processes participating in PAH sensing and defense, (2) explore the PAH accumulation potential and differences or similarities in the response induced by different PAH contaminations based on a component-based approach and a natural-mixture approach, and (3) integrate transcriptomics and metabolomics data to elucidate the defense-related regulations and adaptive mechanisms of *E. purpurea* under PAH exposure. This study will help us comprehend the molecular mechanism of *E. purpurea* against different PAH contaminations.

## 2. Results

### 2.1. Growth and PAH Accumulation

**Figure 1:**
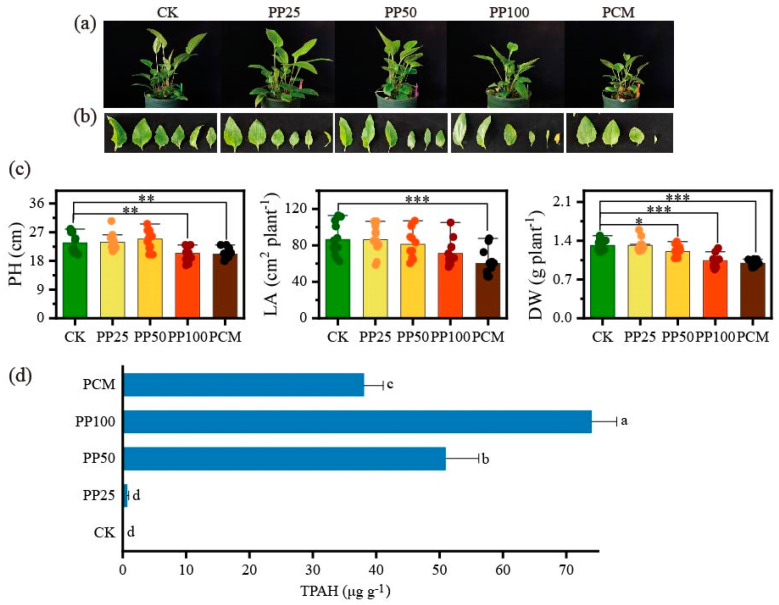
Morphological observation (**a**,**b**), growth (**c**), and PAH accumulation (**d**) of *E. purpurea* upon PAH exposure. PH, plant height; LA, total leaf area; SDW, shoot dry weight; TPAH, total concentration of polycyclic aromatic hydrocarbon; CK, PAHs-free control; PP25, PP50, PP100, defined PHE and PYR at 25 mg kg^−1^, 50 mg kg^−1^, and 100 mg kg^−1^ for each one, respectively; PCM, natural PAH mixture from oilfield site. *, **, and *** represent significance at 0.05, 0.01, and 0.001 test levels, respectively. Different lowercase letters in d indicate significant differences between treatments at *p* < 0.05 (LSD).

The exposure of PHE mixed with PYR at a low level (25 mg kg^−1^) had no remarkable effect on the phenotype, plant height (PH), total leaf area per plant (LA), and shoot dry weight (SDW) of *E. purpurea* plants (Figure 1a–c). Notably, the exposure of PHE mixed with PYR at a high level (100 mg kg^−1^) resulted in small leaves, leaf senescence, and stunted growth in terms of PH (*p* < 0.01) and SDW (*p* < 0.001). Similarly, in PCM (natural mixture of PAH sandwiched with farm soil) treatment, when the plants were exposed to the natural mixture of PAHs derived from aged petroleum-contaminated soils, a significant decrease in growth was observed with respect to the PAH-free control (CK, *p* < 0.001). These results indicated that *E. purpurea* plants showed a clear resistance to PAH exposure at low levels but sensitivity to increased concentrations and complex PAH mixture. However, the highest total concentration of 200 mg kg^−1^ was not acutely lethal for *E. purpurea*. Considering the accumulation of PAH in the leaves of *E. purpurea* (Figure 1d), compared with CK and PP25 (25 mg kg^−1^ PHE+PYR) treatments, in which PAHs were not found or traced, the TPAH significantly increased when the PAH concentration increased in PP50 (50 mg kg^−1^ PHE+PYR) and PP100 (100 mg kg^−1^ PHE+PYR). The PCM treatment exhibited lower total PAH concentration (TPAH) compared to PP25 and PP100. In PP25, no significant difference was observed in morphological and growth characteristics. Therefore, PP50, PP100, and PCM treatments were selected for further transcriptomic and metabolomic analysis.

### 2.2. Overview of RNA Sequencing, Functional Annotation, and Identification of DEGs

**Figure 2:**
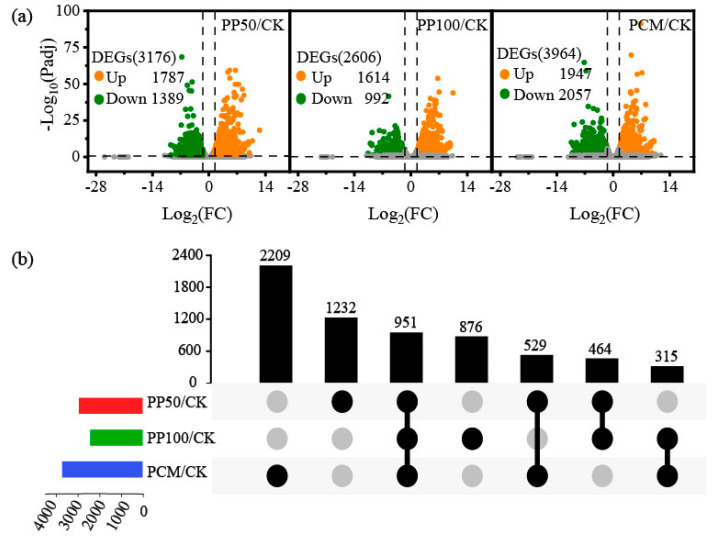
Analysis of DEGs in *E. purpurea* leaves upon PAH exposure (**a**) and upset plot showing shared and unique DEGs across three groups (**b**). Abbreviations of groups are indicated in Figure 1.

An overview of RNA-Seq data and a summary of the length distribution of the transcripts and unigenes are presented in Table S1 and S2, which suggest the high quality of RNA-seq data. Totally, 56,161 (61.54%) unigenes were successfully matched to at least one of the databases according to a comprehensive annotation, and 36,541 (94.49%) annotated unigenes matched five species, of which the most significant homology species was *Helianthus annuus* (L.) (79.22%) (Figure S1). The total numbers of differentially expressed genes (DEGs) identified across all groups varied from 3176 with PP50/CK, 2606 with PP100/CK, to 3964 with PCM/CK. The numbers of up-regulated genes (1787 in PP50/CK and 1614 in PP100/CK) were higher than those of the down-regulated ones (1389 in PP50/CK and 992 in PP100/CK), which showed that the majority of genes were switched on after exposure to PHE and PYR in *E. purpurea* (Figure 2a). Compared with those in the PP50/CK and PP100/CK groups, a larger set of genes in the PCM/CK group showed a response. In addition, the upset plot analysis showed a relatively larger overlap between the three groups with 951 identical DEGs. Meanwhile, 1308 DEGs were shared by at least two but not all groups, and 4317 DEGs were not shared at all (Figure 2b).

### 2.3. Overview of Metabolomic Data and Identification of DAMs

**Figure 3:**
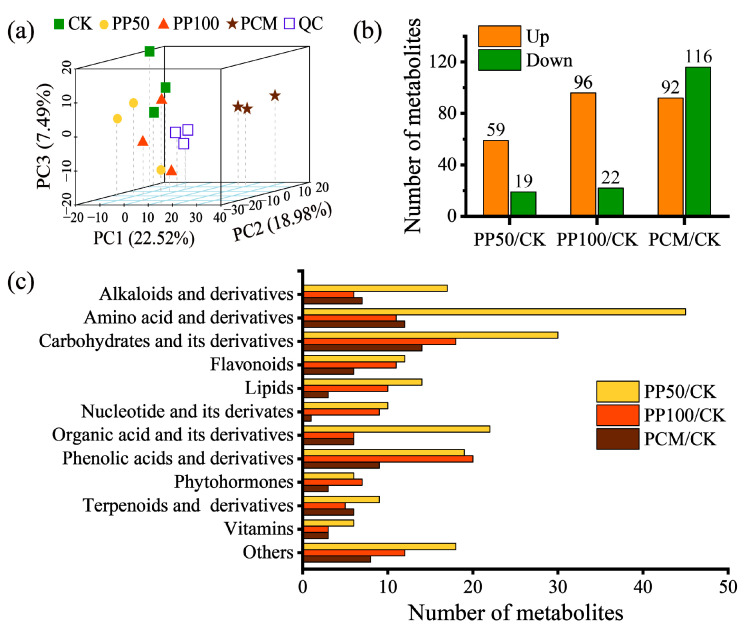
The PCA score plot based on metabolomic data (**a**), total number (**b**), and classification (**c**) of DAMs in *E. purpurea* leaves upon PAH exposure. QC, quality control samples. Abbreviations of groups are indicated in Figure 1.

The good repeatability and instrumental stability of metabolite detection were confirmed by the PCA score plot, which showed that the three quality control samples were overlaid (Figure 3a). Among identified metabolites, 78, 118, and 208 differentially accumulated metabolites (DAMs) were observed in the three comparison groups, and of these, amino acids, carbohydrates, and their derivatives were the first predominant chemical classes (Figure 3b,c). Furthermore, considering the secondary metabolites detected here, PAH treatments affected metabolites predominantly belonging to the class of phenolic acids, flavonoids, and their derivatives. These results demonstrated that PAH exposure strongly affects the metabolite profiles of *E. purpurea*, which was associated with PAH exposure levels.

### 2.4. GO Classification

**Figure 4:**
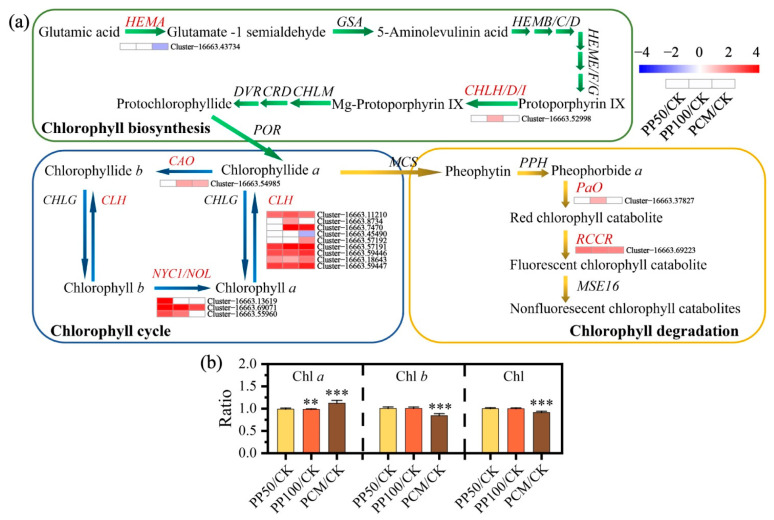
Pathway of chlorophyll metabolism and gene expression (**a**) and chlorophyll contents (**b**) in *E. purpurea* leaves upon PAH exposure. Abbreviations: CAO, geranylgeranyldiphosphate reductase; CHLH/D/I, Mg-chelatase; CLH, chlorophyllase; HEMA, glutamyl-tRNA reductase; NYC1/NOL, chlorophyllide a oxygenase; PaO, pheophorbide a oxygenase; RCCR, red chlorophyll catabolite reductase. **, and *** represent significance at 0.01, and 0.001 test levels, respectively. For more details, see the text. Abbreviations of groups are indicated in Figure 1.

According to GO enrichment analysis, a total of 8 enriched terms in biological process (BP) and 5 enriched terms in molecular function (MF), including 4 sub-categories, namely, “chlorophyll catabolic process”, “pigment catabolic process”, “porphyrin-containing compound catabolic process”, and “tetrapyrrole catabolic process”, were shared between PP100/CK and PCM/CK groups (Figure S2 and Table S3). Totally, 17 genes encoding seven enzymes involved in the chlorophyll metabolic pathway were annotated (Figure 4a). Among these, 1, 3, and 9 genes encoding enzymes related to the chlorophyll cycle, namely, chlorophyllide a oxygenase (CAO), Chl b reductase (non-yellow coloring, NYC1/NOL), and chlorophyllase (CLH), were identified, and most of them were up-regulated under PAH exposure. In addition, the amount of Chla increased, and that of Chlb decreased in PCM, which indicated the enhanced interconversion between Chla and Chlb under complex PAH exposure (Figure 4b). The up-regulated expression of two genes associated with chlorophyll degradation, including Cluster-16663.37827, which encodes pheophorbide a oxidase (PAO) and Cluster-16663.69223, which encodes red Chl catabolite reductase (RCCR), was associated with low Chl level in the PCM leaves (Figure 4b).

### 2.5. KEGG Pathway Analysis

**Figure 5:**
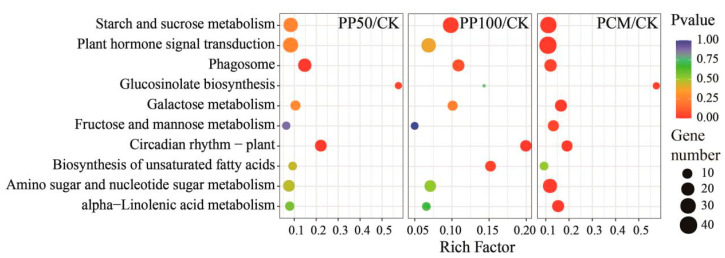
KEGG enrichment analysis of DEGs in *E. purpurea* leaves upon PAH exposure. Bubble size and color correspond to the gene number and Q value enriched in the pathway. The rich factor indicates the ratio of the number of DEGs mapped to a certain pathway to the total number of genes mapped to this pathway. Abbreviations of groups are indicated in Figure 1.

**Figure 6:**
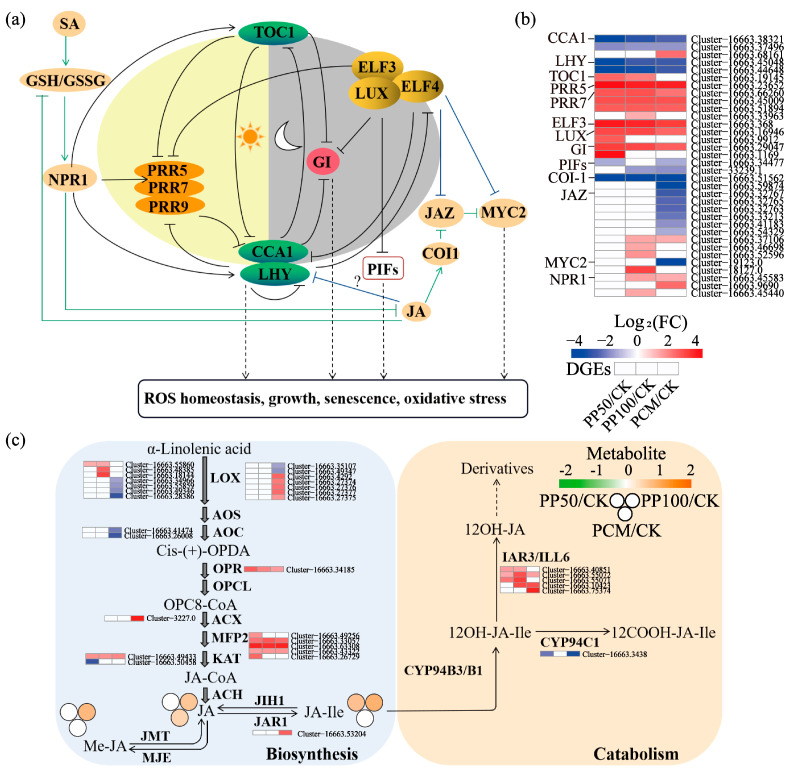
Putative and simplified working model of the circadian clock and its crosstalk with the JA pathway (**a**), expression of associated genes (**b**), and gene expression and metabolite accumulation in JA biosynthesis and catabolism pathways (**c**) in *E. purpurea* leaves upon PAH exposure. The circadian rhythm network was modified from the works Wang et al. (2018) [27] and Zhang et al. (2019) [28]. Scheme of JA biosynthesis and catabolism made based on the works of Huang et al. (2017) [29] and Delgado et al. (2021) [30]. For more details, see the text. Abbreviations of groups are indicated in Figure 1.

The KEGG enrichment analysis showed that 40, 58, and 176 DEGs were significantly enriched in 3, 3, and 9 KEGG pathways, respectively, in the PP50/CK, PP100/CK, and PCM/CK groups (Table S4). The pathway of “circadian rhythm of plant”, which was co-enriched between three groups, and the pathways of “plant hormone signal transduction” and “starch and sucrose metabolism”, which showed the top two largest numbers of DEGs, were selected to perform the subsequent analysis (Figure 5). We identified and annotated nine key components (Figure 6a), including circadian clock-associated 1 (CCA1), timing of cab expression 1 (TOC1), late elongated hypocotyl (LHY), pseudo-response regulator 5/7 (PRR5/7), early flowering 3 (ELE3), lux arrhythmo (LUX), phytochrome interacting factor 4 (PIF4), and gigantea (GI), which are involved in the “circadian rhythm of plant”. The genes encoding CCA1, LHY, and PIF4 were down-regulated, and other genes showed up-regulated expression patterns in response to PAH exposure (Figure 6a,b). The genes in jasmonic acid (JA) and salicylic acid signaling pathways, which are in crosstalk with the circadian clock, were also identified, and most of them in JA were significantly downregulated in three groups, including coronatine insensitive 1 (COI1)-encoding gene, JA ZIM Domain proteins (JAZ)-encoding gene and transcription factor (MYC2). Meanwhile, in SA, genes encoding non-expressor of pathogenesis-related gene 1 (NPR1) were up-regulated. More genes were coregulated in PCM than PP50 and PP100. We also identified three metabolites, namely, JA, JA-isoleucine conjugate (JA-Ile), and methyl JA (MeJA), which showed an increased trend from 1.2- to 2.2-fold (Log2FC), especially under PHE and PYR exposure at 100 mg kg^−1^, which means an enhancement of JA biosynthesis in *E. purpurea* leaves after PAH exposure (Figure 6c). In concert with this, most genes in the JA synthesis pathway were up-regulated in response to PYR and PHE exposure. Together with JA biosynthetic genes, genes in JA-catabolic pathways, namely, JA-Ile oxidation and JA-Ile deconjugation, were coregulated differently to maintain JA homeostasis in response to PAH exposure.

Furthermore, together with the pathway of “starch and sucrose metabolism”, which was enriched in PP100 and PCM groups, two pathways, namely, “galactose metabolism” and “fructose and mannose metabolism”, were enriched in the PCM/CK (Figure S3). In response to PAH exposure, a majority of genes (27 out of 32 in PP50, 39 out of 48 in PP100, and 37 out of 57 in PCM) encoding critical enzymes involved in the three pathways were up-regulated; these enzymes included alpha/beta-glucosidase (malZ) and β-fructofuranosidase (INV) (Figure S3). Consistent with the gene expression, 7, 7, and 12 DAMs classified as carbohydrates and derivatives, including galactose 1-phosphate, D-glucose 1-phosphate, galactinol, melibiose, sucrose, cellobiose, trehalose, trehalose 6-phosphate, maltose, isomaltose, D-fructose 6-phosphate, and D-mannose 6-phosphate, were mapped to three of three pathways in PP50, PP100, and PCM, respectively. In addition, the abundance of these metabolites increased more under PP100 than PP50. In contrast to more metabolites accumulated in PP50 and PP100 in a concentration-dependent manner, more metabolites were depleted in PCM.

### 2.6. Phenolic DAMs

**Figure 7:**
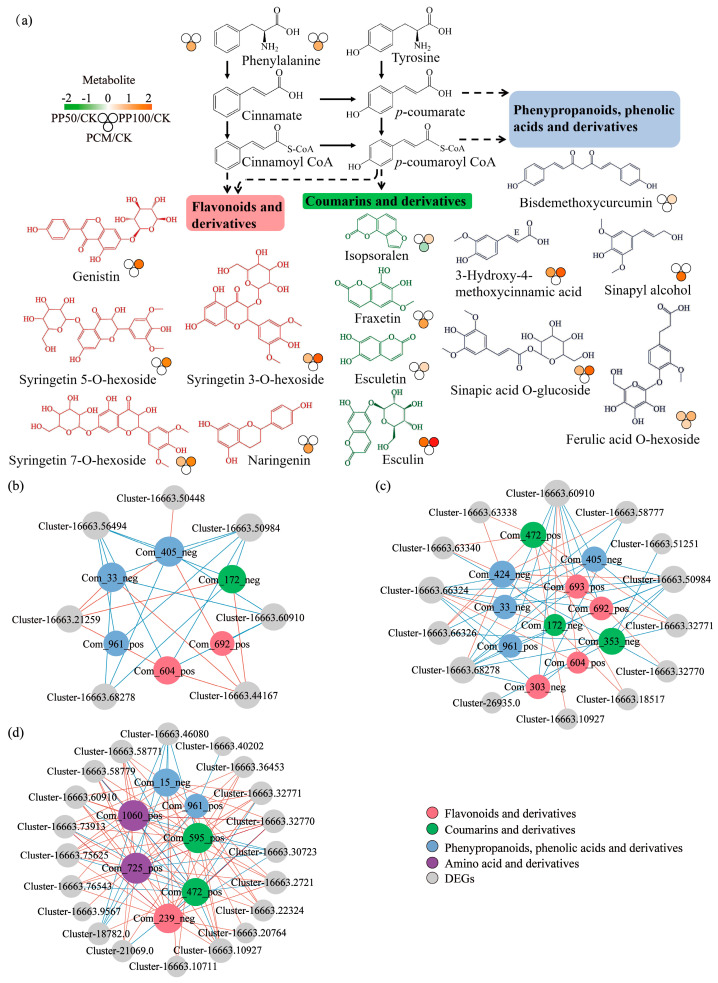
Metabolite accumulation (**a**) and co-expression analysis of metabolites and genes (**b**–**d**) in the phenylpropanoid pathway in *E. purpurea* leaves upon PAH exposure. In a, the identified DAMs with increased levels were categorized into three classes. In (**b**–**d**), a network was constructed for PP50, PP100, and PCM groups, respectively. Color nodes represent DAMs, and grey nodes represent DEGs. Red or blue edges represent positive or negative correlations, respectively. Abbreviations of groups are indicated in Figure 1.

According to the results of metabolomics, phenolic compounds showed the most dramatic alterations in leaves, and their change was explained detailedly (Figure 7a). The main accumulated phenolic compounds produced via the phenylpropanoid pathway in *E. purpurea* leaves were flavonoids and their derivatives (e.g., naringenin, genistin, and syringetin 3/5/7-O-hexoside), coumarins and their derivatives (e.g., esculetin, esculin, fraxetin, and isopsoralen), and phenypropanoids, phenolic acids, and their derivatives (e.g., bisdemethoxycurcumin, sinapic acid O-glucoside, sinapyl alcohol, 3-hydroxy-4-methoxy-cinnamic acid, and ferulic acid O-hexoside) (Figure 7a). Esculin, syringetin 3-O-hexoside, and 3-Hydroxy-4-methoxycinnamic acid were significantly increased by 3.0-, 1.5-, and 2.2-fold in the PP50, and 5.4-, 3.8-, and 3.7-fold in PP100, respectively. Meanwhile, sinapyl alcohol significantly increased by 3.4-fold in the PCM. To determine the association between the metabolites and genes that derived flavonoid and phenylpropanoid pathways, we constructed networks based on the correlation analysis (Figure 7b–d). In PP50, PP100, and PCM, 77, 91, and 119 related pairs and several key DEGs were identified, and they mainly included chalcone synthase (CHS), which is involved in the flavonoid biosynthesis pathway, beta-glucosidase (BGLU), caffeoyl-CoA O-methyltransferase (CCoAOMT), and caffeic acid 3-O-methyltransferase (COMT), which are involved in the phenylpropanoid biosynthesis pathway.

### 2.7. Association Analysis of Metabolome and Transcriptome

Association analysis of KEGG based on metabolome and transcriptome indicated that “starch and sucrose metabolism” was mainly enriched in all three treatments (Figure S4a–c), which was consistent with the results obtained in Figure 4. To explore the links among DEGs, DAMs, and growth properties, the Mantel test was used (Figure S4d). The results indicated that both TPAH (Mantel’s r = 0.546, 0.610, *p* < 0.05) and SDW (Mantel’s r = 0.616, 0.710, *p* < 0.05) showed a stronger correlation with DEGs and DAMs, respectively. Furthermore, Chla was significantly correlated with DEGs and DAMs; LA was correlated with DAMs. These results support that metabolite accumulations and transcription regulations contribute to the growth response of *E. purpurea* to PAH exposure.

## 3. Discussion

### 3.1. Responses of Growth and Chlorophyll Metabolism in E. purpurea Leaves to PAH Exposure

The advantages of phytoremediation have been mentioned previously; however, the most considerable disadvantages are the toxicity of PAH for the plants over a certain concentration threshold, which can limit the phytoremediation efficiency [31]. Tolerance of plants to contaminants is an essential criterion for successful phytoremediation [32,33]. Our results showed that *E. purpurea* exhibited a tolerant phenotype under PAH treatments (as a mixture of PHE and PYR) within the tested dosages, especially at lower dosages of 50 mg kg^−1^ and 100 mg kg^−1^ (Figure 1). Conversely, *E. purpurea* was less resistant to the natural PAH mixture in PCM treatment and exhibited reduced growth and decreased physiological parameters (Figure 1 and Figure 4). Supporting this result, PCC analysis among leaf parameters also showed that TPAH in the leaf was negatively correlated with SDW, LA, and Chla (Figure S5). In this work, members of genes involved in the chlorophyll cycle, such as *CAO*, *NYC1/NOL,* and *CLH*, displayed similar expression patterns and were significantly up-regulated and then promoted the cycle of chlorophyll in *E. purpurea* leaves when PAH-exposed (Figure 4). PaO is a key enzyme for chlorophyll breakdown, and it catalyzes open the porphyrin ring of pheophorbide to form red chlorophyll metabolite; then it is converted into a primary fluorescent chlorophyll metabolite by another key enzyme, RCCR [34]. Here, the high expression of genes encoding PaO and RCCR showed similar trends to those observed in Chl content analysis (Figure 4). Hence, the low chlorophyll content in PCM could be explained partly by the promotion of the chlorophyll cycle and degradation under exposure to the natural PAH mixture.

### 3.2. Effects on Circadian Rhythm and Hormone Signal Transduction in E. purpurea Leaves

In *A. thaliana*, the core oscillator of the circadian clock mainly consists of CCA1, LHY, TOC1, PRR9/7/5, ELF3, ELF4, LUX, and GI, which comprises three interlocking negative feedback loops (central/morning/evening loop) [27,28,35,36]. Given that the clock transcriptional network in *A. thaliana* is partly conserved among angiosperms [37], the expression of associated genes in *E. purpurea* was characterized (Figure 6). After PAH exposure, circadian genes, including *CCA1*, *LHY*, and *PIF,* were downregulated, whereas other genes, such as *ELF3*, *LUX*, *GI*, *PPR5*, and *PPR7,* showed up-regulated expression patterns. To our best knowledge, this work provides the first observation of circadian regulation in plants under PAH exposure, and the results may help in elucidating the function of circadian clock regulation in the defense responses of plants to PAH contamination. As another component of the circadian clock molecular system, circadian output pathways, which include a series of downstream events, can contribute to the ability of plants to adapt to environmental alterations [38,39]. Emerging evidence shows that as key components of the core oscillator, CCA1, PRR7, and ELF3 are associated with plant growth and leaf senescence [36,40]. Therefore, we concluded that during PAH phytoremediation, the reduced plant growth and enhanced leaf senescence in *E. purpurea* leaves are modulated by the circadian clock (Figure 1, Figure 4 and Figure 6). In addition, the circadian clock also functions as a master regulator in phytohormone biosynthesis and signaling [40]. In this concern, JA biosynthesis, homeostasis, and signaling are regulated by the circadian clock, whereas, in a feedback manner, JA also participates in the regulation of circadian speed [41,42]. Furthermore, JA can induce secondary compounds biosynthesis and participate in the plants' adaptation to environmental stresses [29,43,44,45]. Here, the expressions of genes associated with the pathways of JA and the circadian network were the most affected, and the biosynthesis of hormones, such as JA, JA-Ile, and Me-JA, was remarkably enhanced (Figure 4 and Figure 5), which contributed to the ability of *E. purpurea* to adapt to PAH stress.

### 3.3. Effects on Sugar Metabolism and Secondary Metabolism in E. purpurea Leaves

In plants experiencing different environmental stress conditions, saccharide accumulation in the cytoplasm is an important mechanism for maintaining osmotic balance [46,47]. Moreover, by providing more energy resources to the plant, carbohydrates can improve the synthesis of particular anti-stress compounds, such as secondary metabolites, for survival under adverse conditions [48]. An earlier study confirmed that galactose metabolism is the most remarkable metabolic pathway disrupted by BaP and PYR exposure in *Zea mays* (L.) leaves [25]. Equally, we expanded on those findings and confirmed that most DEGs, i.e., *malZ* and *INV*, in galactose metabolism, were significantly up-regulated in response to PAH exposure, either as a defined or natural mixture (Figure S3a). Sucrose, which is the basic stored form of saccharides, can function both in plant growth and signal transduction as well as stress mitigation [47]. In the present study (Figure S3), the plants subjected to PAH exposure presented a significant accumulation of sucrose, together with galactinol and melibiose, which provided the *E. purpurea* plant with high energy levels and protected it against PAH stress. This was confirmed by association analysis of KEGG pathways (Figure S4), which also indicated that the “starch and sucrose metabolism” pathway was mainly enriched based on metabolomic and transcriptomic data.

The Mantel test revealed that TPAH was strongly correlated to DEGs and DAMs, which indicated that PAH concentration is tightly associated with gene regulation and metabolite accumulation in *E. purpurea* leaves and may explain the metabolic responses caused by PAH exposure (Figure S4). In this concern, the accumulation of several phenolic metabolites, such as phenolic acids, flavonoids, and coumarins, which were produced via the phenylpropanoid pathway, was observed (Figure 3 and Figure 7). Furthermore, the regulation of phenylpropanoid pathway genes (*COMT*, *CCoAOMT*, *BGLU*, and *CHS*), which were associated with the metabolism of phenolic acids and synthesis of coumarins and flavonoids, was found (Figure 7). Phenolic secondary metabolites have different functions in plant growth and stress response [49,50,51]. Spinedi et al. (2021) [6] and Li et al. (2021) [21] demonstrated that the up-regulation of genes in flavonoid biosynthesis and the accumulation of flavonoid compounds could contribute to the antioxidant activity against anthracene toxicity in *Marchantia Polymorpha* (L.) or is the key to enhancing tolerance to PHE stress in *A. thaliana*, respectively. Among numerous structural modulations, glycosylation can alter the homeostasis of polyphenols and glycosidic phenols with antioxidant activity and function in plant defense response [52]. In this study, glycosidic flavonoids, such as syringetin 3/5/7-O-hexoside, accumulated higher contents in *E. purpurea* leaves of PP50 and PP100 compared with the CK (Figure 7). Although our understanding of the roles of secondary metabolites and their derivatives in PAH phytoremediation remains limited, the accumulation of phenolic secondary metabolites and the regulation of phenylpropanoid and flavonoid biosynthesis pathways partially function in the biochemical adaptability of *E. purpurea* after PAH exposure. However, the ecological aspect of invasive exotic plant species [53] needs to be considered in future research in order to achieve sustainable phytoremediation of PAHs using *E. purpurea*.

## 4. Materials and Methods

### 4.1. Soil Preparation, Experimental Design, and Plant Cultivation

Two soil types were used in this study. One (farm soil) was clay loam soil with no history of contamination and contained only background levels of PAH or no detectable PAH. This soil was collected from an experimental farm near Shenyang City, Liaoning Province, China, at a depth of approximately 20 cm. The other (oilfield soil) was aged PAH-contaminated soil obtained from Daqing Oilfield, which has been operating for more than 30 years in Daqing City, Heilongjiang Province, China. After collection, the soils were air-dried and homogenized by sieving to remove stones and residual plant materials. Table S5 summarizes the basic characteristics of the soil samples. Five treatments based on a component-based approach and a natural-mixture approach were set up as follows: farm soil without PAHs (CK); farm soils spiked with PHE and PYR mixtures at 25 (PP25), 50 (PP50), and 100 mg kg^−1^ (PP100) for each one; oilfield soil containing natural mixture of PAH sandwiched with farm soil (PCM). The selected PAH concentration was the range described in previous studies [25,54]. In the PP25, PP50, and PP100 treatments, 10% total quantity of farm soils was spiked with highly-pure PHE and PYR in acetone. Afterward, the soils were left under the fume hood to evaporate any traces of acetone. Then, the spiked soils were thoroughly mixed with uncontaminated farm soils to obtain initial total PAH concentrations of 50, 100, and 200 mg kg^−1^. The sum of seven PAHs concentrations in the oilfield soil was about 150 mg kg^−1^, and chrysene (CHR, 65.3%), PHE (20.6%), and fluoranthene (5.7%) were the major species (analysis method is shown in Section 2.3). The aged oilfield soil was sandwiched with uncontaminated farm soil in accordance with the method of Dai et al. (2020) [55] for the PCM treatment. The contaminated soil in the PCM treatment was diluted to a PAH concentration of about 50 mg kg^−1^ through the addition of uncontaminated farm soil. 

The experimental soil was placed in 1.5 L pots with an underpan to avoid chemical and soil loss when watering. Then, 2-month-old *E. purpurea* seedlings with similar appearances, plant height (PH), and leaf quantity were selected and transferred to these pots for the phytoremediation experiment stretched over a period of 2 months (60 d). After transplanting, the pots with twelve replicates of each treatment were cultivated in a growth chamber (a 16/8 h light/dark cycle and temperatures of 18 °C–27 °C.) following the method of Heidari et al. (2018) [11]. Each pot was irrigated regularly to avoid excessive drainage. After 2 months of phytoremediation, *E. purpurea* plants were collected. Then, the plants were divided into two parallel groups for growth measurement (twelve biological replications), PAH analysis, metabolome analysis, and transcriptome sequencing (three biological replications). After collection, the leaf samples were immediately frozen and then stored at −80 °C until subsequent metabolite identification, transcriptome sequencing, and quantitative real-time polymerase chain reaction (qRT-PCR) validation.

### 4.2. Growth Measurement

The plant height (PH), total leaf area per plant (LA), Chlorophyll a (Chla), Chlb, and total Chl contents in leaves were measured in accordance with the method of Sun et al. (2018) [56]. Afterward, the shoot dry weight (SDW) was determined via oven-drying at 80 °C until constant weight.

### 4.3. PAH Extraction and Analysis

The PAH concentration in leaf samples was determined in accordance with the method described by Wang et al. (2012) and Chen et al. (2019) with modifications [32,57]. Briefly, 5 g each of the leaf sample and quartz sand (blank sample) were placed in a centrifuge tube with 10 mL dichloromethane and 20 μL 200 mg mL^−1^ deuterated triphenyl solution as the recovery standard. Subsequently, the tube was ultrasonicated thrice for 1 h each at 35 °C. The extracts were combined, concentrated with gentle nitrogen blowing to 1 mL, and then passed through a preconditioned magnesium silicate SPE column (1000 mg 6 mL^−1^) with 11 mL 1:1 (*v*/*v*) elution of dichloromethane and n-hexane. After filtration, the extracts were re-concentrated with gentle nitrogen blowing, transferred to a sample vial containing 50 μL 20 μg mL^−1^ intermediate standard solutions, and diluted with n-hexane to 1 mL. The internal standard was the mixture of deuterated PAHs, including Acenaphthylene d10, PHE d10, CHR d12, and perylene d12 (J&K Scientific Inc.; Beijing, China). A gas chromatograph mass spectrometer (HP6890-5975B, Agilent Co.; Santa Clara, CA, USA) equipped with an HP-5MS column was used for the quantification of target PAH concentrations in the extracts.

### 4.4. Transcriptomic Sequencing and qRT-PCR

RNA isolation was carried out following a method described in our previous work [58], and RNA-seq was carried out using an Illumina HiSeq 6000 system (Illumina, San Diego, CA, USA) at Novogene Biotech (Beijing, China). The sequenced reads (NCBI SRA accession number: PRJNA905488) were filtered by the removal of low-quality reads for subsequent analysis. Transcriptome assembly sequences were annotated in seven databases, including NR, NT, KO, Swiss-Prot, Pfam, KOG, and GO. Differentially expressed genes (DEGs) were identified at a cutoff of |Log2FC)| > 1 (false discovery rate and adjusted *p* < 0.05). Ten DEGs with different putative functions were selected to confirm the RNA-Seq results by performing qRT-PCR following our previous work [58]. The employed primers are summarized in Table S6, and the correlation between RNA-seq and qRT-PCR data is presented in Figure S6.

### 4.5. Metabolomic Analysis

The widely targeted metabolomic analysis was performed using the high-performance liquid chromatography-tandem mass spectrometry with an ExionLC™ AD system and an Xselect HSS T3 column (150 × 2.1 mm^2^, 2.5 μm) coupled to a QTRAP^®^ 6500+ mass spectrometer (SCIEX) at Novogene Biotech (Beijing, China) following a method described in our previous work [57]. Compound Discoverer software was used to search the online libraries of mzCloud, mzVault, and ChemSpider to identify the metabolites. After that, the differentially accumulated metabolites (DAMs) were screened considering variable importance in the projection > 1, *p* < 0.05, and |Log2FC)| > 1 and then subjected to KEGG enrichment analysis.

### 4.6. Data and statistical analysis

One-way analysis of variance was carried out to test the effect of PAH treatments on growth parameters using SPSS 25.0 (IBM Co.; Armonk, NY, USA). A volcano plot was performed to filter DEGs based on the −Log10p values of genes. Principal component analysis (PCA) was performed to visualize the differences among samples using the Vegan package in R (v4.0.3, https://www.r-project.org/ (accessed on 9 March 2023)). Heatmaps were generated to identify the expression profiles of DEGs or alterations in DAMs under treatments using the pheatmap package in R. To integrate transcriptomic and metabolomic data, the Pearson correlation coefficients (PCCs) analysis among growth parameters and partial Mantel test between growth parameters, DEGs and DAMs were performed using the Vegan package in R (9999 permutations), respectively.

## 5. Conclusions

Integrating transcriptomic data with metabolomic data, we concluded that PAH exposure induced the regulation of multiple pathways and processes, including chlorophyll cycle and degradation, circadian rhythm, JA biosynthesis and signaling, and starch and sucrose metabolism. The accumulation of metabolites, especially secondary metabolites produced via the phenylpropanoid pathway (coumarins and flavonoids), is also responsible for the adaptation of *E. purpurea* to PAH contamination. The identified specific gene and metabolite targets are associated with primary processes set up to sense PAH as well as with molecular and metabolic mechanisms to tolerate PAH exposure in *E. purpurea*. Collectively, this novel information opens a new avenue for the mechanism research of PAH phytoremediation, which will be valuable in the improvement of phytoremediation efficiency with *E. purpurea* via a mechanism-based strategy.

## Data Availability

Sequence data from this work can be found in the NCBI database (SRA data).

## Supplementary Materials

The following supporting information can be downloaded at: https://www.mdpi.com/article/10.3390/ijms241311020/s1. Table S1: Summary of RNA-seq data and quality control result. Table S2: Number and length distribution of the transcripts and unigenes from RNA-seq. Table S3: GO term enrichment analysis of DEGs response to PAH exposure. Table S4: KEGG classifications of DEGs in the *E. purpurea* leaf upon PAHs exposure. Table S5: The basic chemical characteristics of the test soil. Table S6: Primers used for qRT-PCR. Figure S1: Unigene function annotation statistics (a), species distribution (b), and similarity distribution (c) based on *E. purpurea* transcriptome. Figure S2. Annotated biological functions obtained by GO enrichment analysis of *E. purpurea* upon PAH exposure. Figure S3: Gene expression and metabolite accumulation changes in the sugar metabolism pathways in *E. purpurea* leaves upon PAH exposure. Figure S4: Integrated analysis of metabolomic and transcriptomic data of *E. purpurea* leaves under PAH exposure. Figure S5: Correlation between RNA-seq and qRT-PCR data.
