# Supplementary material for: Molecular Defensive Mechanism of Echinacea purpurea (L.) Moench against PAH Contaminations
Source: Int J Mol Sci. 2023 Jul 3;24(13):11020. doi: 10.3390/ijms241311020 (PMC10341506; doi:10.3390/ijms241311020)
Supplement: Supplementary file 1 [file ijms-24-11020-s001.zip › Figure S1-S5.pdf]

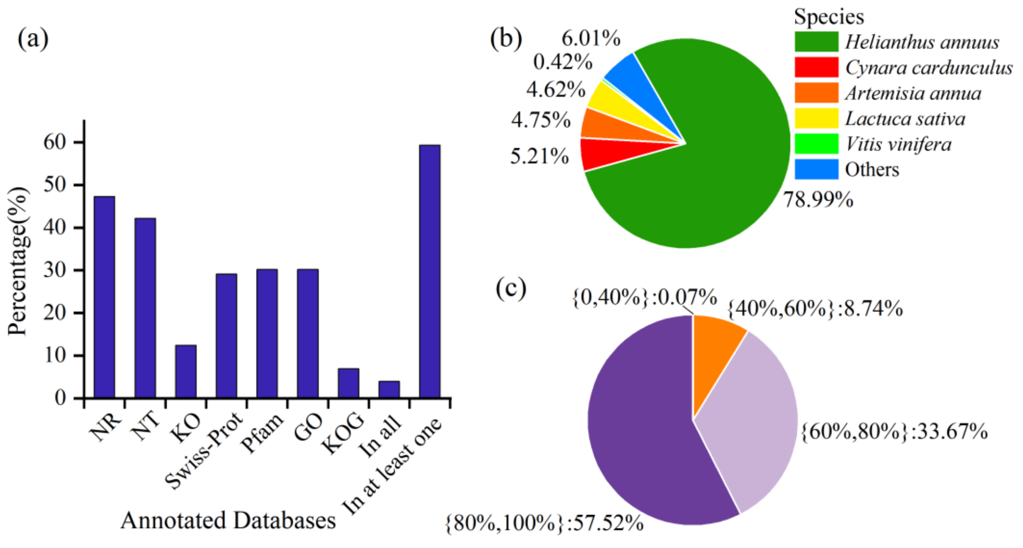

Figure S1. Unigene function annotation statistics (a), species distribution (b), and similarity distribution (c) based on *E. purpurea* transcriptome. NR, NCBI non-redundant protein sequences; NT, NCBI nucleotide sequences; KO, KEGG orthology; Swiss-Prot, a manually annotated and reviewed protein sequence; Pfam, protein family; GO, gene ontology; KOG, euKaryotic orthologous groups.

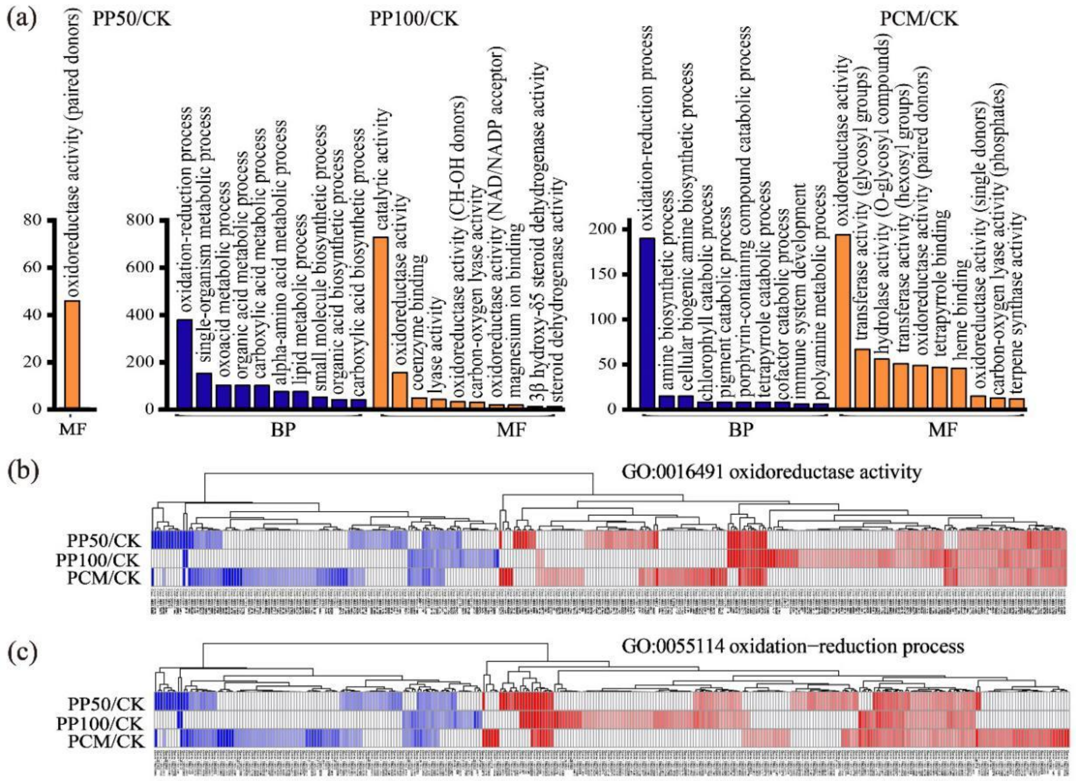

Figure S2. Annotated biological functions obtained by GO enrichment analysis of *E. purpurea* upon PAH exposure. (a) GO functional classification of DEGs. The enriched terms with top 10 gene numbers in each core category were listed. (b and c) Expression profiles of DEGs mapped to GO terms related to "oxidoreductase activity" and "oxidation reduction process", respectively. CK, PAHs free control; PP25, PP50, PP100, defined PHE and PYR at 25 mg kg^{-1}, 50 mg kg^{-1}, and 100 mg kg^{-1} for each one, respectively; PCM, natural PAH mixture from oilfield site.

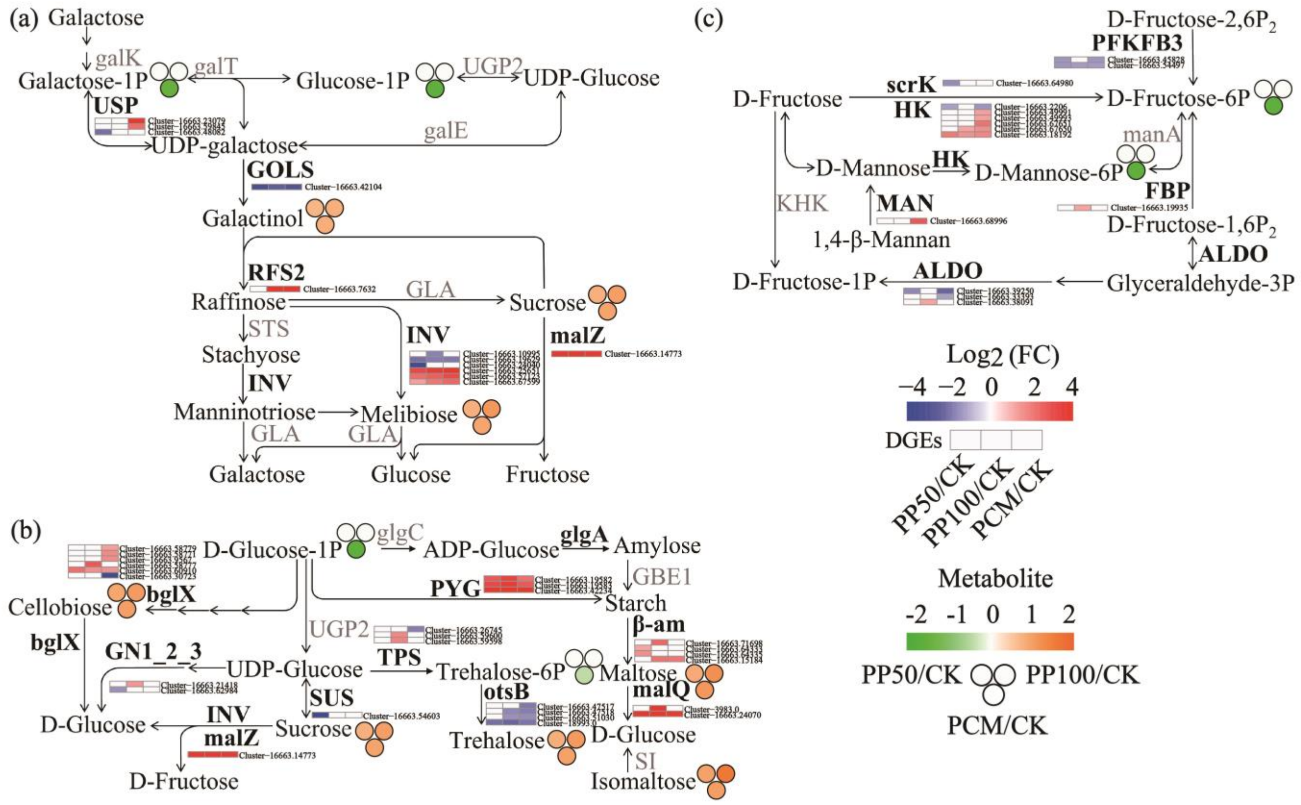

Figure S3. Gene expression and metabolite accumulation changes in the sugar metabolism pathways in *E. purpurea* leaves upon PAH exposure. (a) galactose metabolism, (b) starch and sucrose metabolism, and (c) fructose and mannose metabolism. Abbreviations of key enzymes: ALDO, fructose-bisphosphate aldolase; bglX, beta-glucosidase; FBP, fructose-1,6-bisphosphatase; glgA, starch synthase; GN1_2_3, glucan endo-1,3-beta-glucosidase 1/2/3; GOLS, inositol 3-alpha-galactosyltransferase; HK, hexokinase; INV, beta-fructofuranosidase; malQ, 4-alpha-glucanotransferase; malZ, alpha-glucosidase; MAN, mannan endo-1,4-beta-mannosidase; otsB, trehalose 6-phosphate phosphatase; PFKFB3, 6-phosphofructo-2-kinase/fructose-2,6-biphosphatase; PYG, glycogen phosphorylase; RFS2, raffinose synthase; scrK, fructokinase; SUS, sucrose synthase; TPS, trehalose 6-phosphate synthase/phosphatase; β-am, beta-amylase. Abbreviations of groups were indicated in Figure 2.

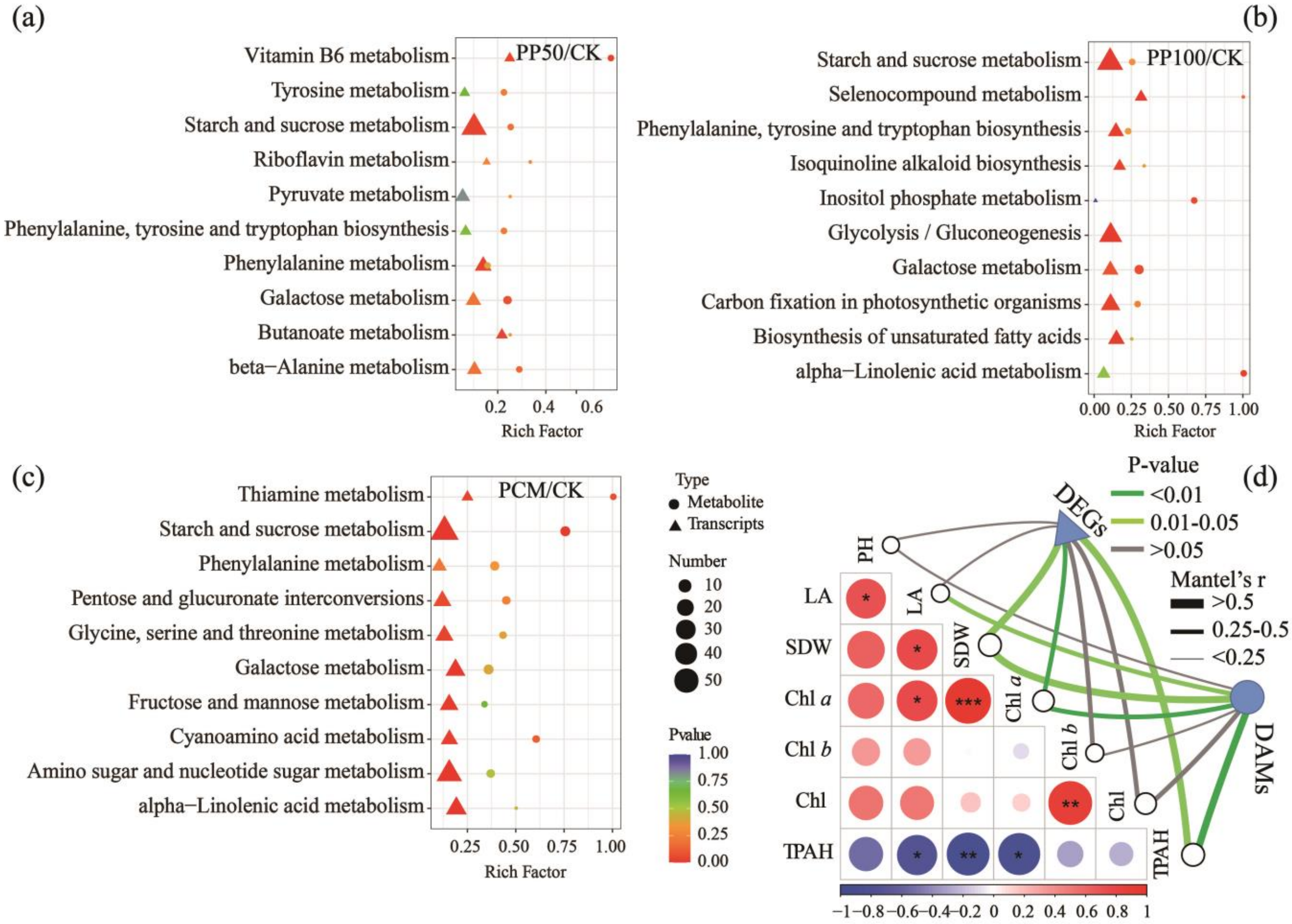

Figure S4. Integrated analysis of metabolomic and transcriptomic data of *E. purpurea* leaves under PAH exposure. (a–c) Association of KEGG pathway in PP25, PP100, and PCM group, respectively. (d) Pearson and Mantel tests of DEGs, DAMs, and leaf traits. Abbreviations were indicated in Figure 2.

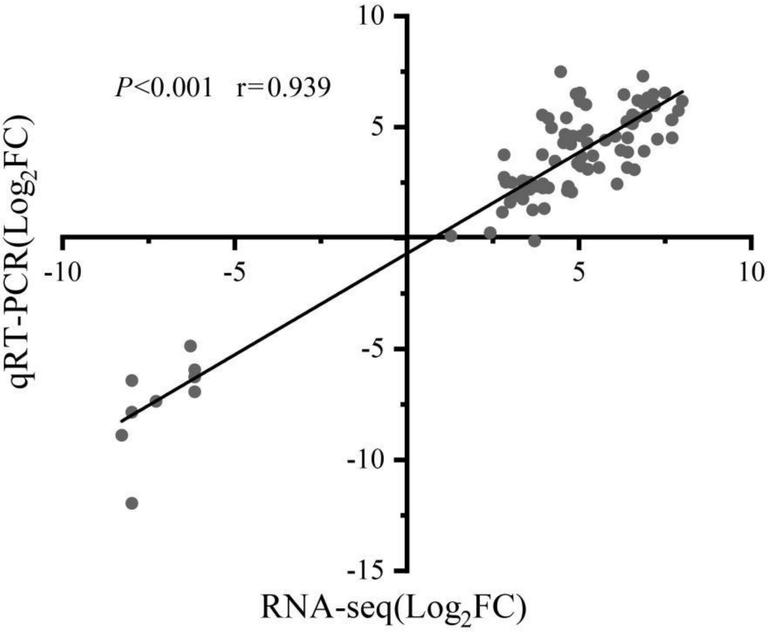

Figure S5. Correlation between RNA-seq and qRT-PCR data. Each RNA-seq expression data was plotted against qRT-PCR data and fitted into a linear regression.
